# Clusterin Plasma Concentrations Are Decreased in Sepsis and Inversely Correlated with Established Markers of Inflammation

**DOI:** 10.3390/diagnostics12123010

**Published:** 2022-12-01

**Authors:** Eray Yagmur, Samira Abu Jhaisha, Lukas Buendgens, Nadezhda Sapundzhieva, Jonathan F. Brozat, Philipp Hohlstein, Maike R. Pollmanns, Ger H. Koek, Ralf Weiskirchen, Christian Trautwein, Frank Tacke, Theresa H. Wirtz, Alexander Koch

**Affiliations:** 1 Institute of Laboratory Medicine, Western Palatinate Hospital, 67655 Kaiserslautern, Germany; 2 Department of Medicine III, RWTH-University Hospital Aachen, Pauwelsstrasse 30, 52074 Aachen, Germany; 3 Division of Gastroenterology and Hepatology, Department of Internal Medicine, Maastricht University Medical Centre (MUMC), 6229 HX Maastricht, The Netherlands; 4 Institute of Molecular Pathobiochemistry, Experimental Gene Therapy and Clinical Chemistry (IFMPEGKC), RWTH-University Hospital Aachen, Pauwelsstrasse 30, 52074 Aachen, Germany; 5 Department of Hepatology and Gastroenterology, Charité Universitätsmedizin Berlin, Campus Virchow-Klinikum und Campus Charité Mitte, 10117 Berlin, Germany

**Keywords:** clusterin, ICU, sepsis, prognosis, organ failure, cellular stress, inflammation, diabetes

## Abstract

Clusterin is a multifunctional protein that is recognized to mediate cellular stress response associated with organ failure, systemic inflammation, and metabolic alterations. The aim of this study was to determine the value of clusterin as a clinical biomarker in critical ill patients with or without sepsis. We analyzed clusterin plasma concentrations in 200 critically ill patients (133 with sepsis, 67 without sepsis) on admission to the medical intensive care unit (ICU). The results were compared with 66 healthy controls. Clusterin plasma concentration was significantly elevated in critically ill patients compared to healthy subjects. Clusterin levels were significantly higher in non-septic ICU patients than in patients with sepsis. Clusterin correlated inversely with routinely used biomarkers of inflammatory response. Furthermore, clusterin levels were higher in ICU patients with pre-existing obesity and type 2 diabetes. Clusterin was not associated with disease severity, organ failure, or mortality in the ICU. This study highlights significantly elevated clusterin levels in critically ill patients, predominantly in non-sepsis conditions, and associates circulating clusterin to inflammatory and metabolic dysfunctions.

## 1. Introduction

The polyfunctional protein clusterin is constitutively expressed and located in a broad range of tissues and body fluids [1]. As a chaperone, secreted clusterin was first associated with Alzheimer’s disease due to its ability to clear misfolded proteins such as β-amyloid and interstitial cellular debris [2,3]. In addition to the attributed neuroprotective role, clusterin has been described as protective in the context of chronic pain, obesity, and cardiac disease [4,5].

In the last years, it has become even more obvious that clusterin exerts highly diversified functions, including the regulation of immune and stress response, apoptosis, and cellular energy metabolism [6,7,8,9]. Thus, clusterin has been described as sensitive to different types of stressors, such as pro-inflammatory, oxidative, or endoplasmic reticulum-associated cell dysfunction. Clusterin modulates the immunological complement system by regulating the formation of the membrane attack complex (MAC) from complement components C5b, C6, and C7. Furthermore, clusterin modulates or reduces pro-inflammatory processes, such as NF-κB-signaling and several cytokines, including transforming growth factor-β (TGF-β), tumor necrosis factor-α (TNF-α), and interleukin-2 (IL-2) [10,11,12,13,14,15].

Three different isoforms of clusterin generated from the same protein precursor have been described: a small nuclear isoform (approx. 49 kDa), a medium-sized isoform remaining in the cytosol and mitochondria, and the larger secreted isoform (approx. 75–80 kDa) [16,17,18,19]. The secreted clusterin is a highly glycosylated 75–80 kDa heterodimer consisting of two disulfide-linked chains of 40 kDa each [20]. After cytoplasmatic release, clusterin can be internalized by cells to participate in intracellular pathways when cell damage or stress requires a protective mechanism to prevent cell death [13,21]. In this context, clusterin expression is elevated in injured arteries due to atherosclerosis [22]. Clusterin removes cholesterol from macrophage foam cells in atherosclerotic lesions [23]. Together with apolipoprotein E and apolipoprotein A-I, clusterin forms HDL cholesterol and promotes its hepatic metabolization [1]. Clusterin is also expressed in adipose tissue [24]. Thus, on the one hand, Clusterin is positively associated with metabolic parameters, such as glycosylated hemoglobin A1c, insulin resistance by HOMA-IR, and fasting C-peptide levels, and has been proposed as a communicator between adipocytes and the liver [25]. On the other hand, bariatric surgery followed by weight loss also results in decreased clusterin expression [26].

In consideration of its cellular, immunologic, and metabolic effects, clusterin has associations to various systemic inflammatory, metabolic, and neurological disorders; and their clinical outcome and phenotype, respectively [3,27,28,29,30,31,32,33].

In a recently published rodent model, De Miguel et al. showed that clusterin was increased in blood plasma in voluntarily exercising mice (so called “runner plasma”) and, when infused into sedentary mice, reduced baseline neuroinflammatory gene expression and brain inflammation [34].

Due to the frequent presence of both inflammatory and metabolic alterations in critically ill patients, it is reasonable to consider clusterin as a beneficial diagnostic tool in terms of disease severity, immunologic modulation, metabolic changes, and prediction of mortality in an intensive care unit (ICU) setting. However, the associations between clusterin and these previously mentioned clinical alterations in ICU patients have not been thoroughly investigated so far. For this reason, we conducted a detailed investigation on the clinical relevance of clusterin in a meticulously documented cohort of ICU patients with and without sepsis.

## 2. Materials and Methods

### 2.1. Selection and Inclusion of Patients

For this observational cohort study, we included patients above the age of 18 admitted to the medical intensive care unit of the Department of Gastroenterology, Digestive Diseases, and Intensive Care medicine of University Hospital RWTH Aachen, Germany from March 2006 to March 2011. Patients were classified into septic and non-septic following the Third International Consensus Definitions for Sepsis and Septic Shock (Sepsis-3) [35], and treatment was performed in accordance with the current guidelines for the treatment of sepsis (Surviving Sepsis Campaign) [36]. Patients referred for follow up after invasive procedures or after elective surgical intervention only were excluded [37]. To assess long-term patient survival, we contacted the patients, their relatives, and/or primary care physicians at 6-month intervals for 2 years after discharge from intensive care [38]. Blood donors were selected as a healthy control group with the following criteria: normal blood count; no biochemical evidence of hepatic or metabolic disease, or viral disease, such as viral hepatitis or HIV [38]. The local ethics committee, in accordance with the ethical standards of the Declaration of Helsinki (reference number EK 150/06), approved our study. Written informed consent was obtained from the patient, his/her spouse, or legal guardian.

### 2.2. Laboratory Analysis of Circulating Clusterin in Patients’ Plasma

Measurement of clusterin concentration was conducted from blood samples obtained prior to the therapeutic treatment of patients. According to the manufacturer’s instructions, each blood sample was centrifuged immediately after collection and the plasma was stored at −80 °C until analysis with a quantitative sandwich enzyme-linked immunosorbent assay (ELISA) (clusterin (human) competitive ELISA, #AG-45A-0013Y, Adipogen AG, Liestal, Switzerland).

### 2.3. Statistical Analysis

An explorative descriptive analysis based on a non-parametric distribution was performed for the statistical evaluation of the parameters. All the quantitative parameters were therefore presented as medians including range, and showed graphically as box-and-whiskers plots. The association between different variables and clusterin was examined using the Spearman rank correlation test. The significance of parameters between the two different groups was assessed with the Mann–Whitney U test. Statistically significant was assumed to be any comparison with a *p*-value less than 0.05. Statistical analyses were carried out using IBM SPSS Statistics (SPSS; Chicago, IL, USA).

## 3. Results

### 3.1. Clusterin Concentrations Are Increased in Critically Ill Patients but Significantly Lower in ICU Patients with Sepsis

**Figure 1:**
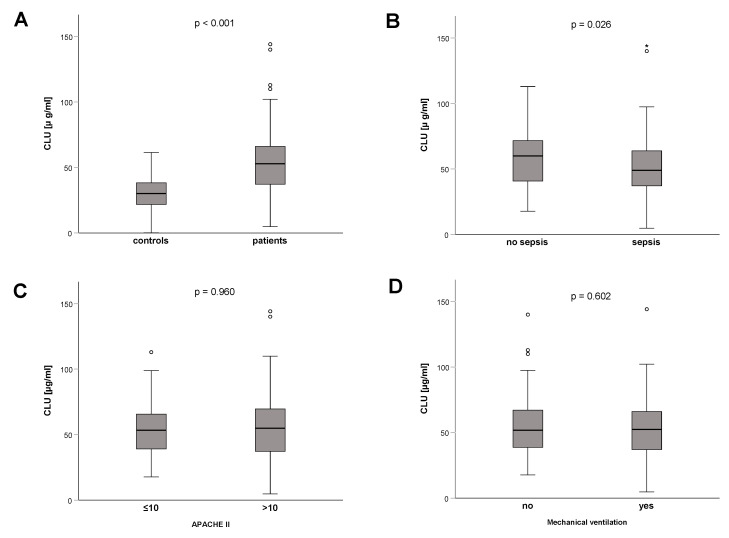
Clusterin levels in critically ill patients. (**A**) Clusterin plasma concentrations are significantly elevated in critically ill patients compared with healthy controls. (**B**) Clusterin levels are significantly lower in ICU patients with sepsis compared to ICU patients without sepsis. (**C**) High disease severity, as defined by an APACHE-II score above 10, is not associated with altered plasma clusterin. (**D**) The need of mechanical ventilation was not associated with clusterin levels at ICU admission. *p*-values (U-test) are given in the figure. Circle depicts outlier. * depicts extreme outlier.

Clusterin plasma concentrations were significantly increased upon the ICU admission of 200 critically ill patients (median 52.9 µg/mL, range 4.8–144.1 µg/mL; Table 1) compared with 66 healthy individuals (median 30.1 µg/mL, range 0.1–61.4 µg/mL, *p* < 0.001; Figure 1A). Nevertheless, we did not observe this increase in clusterin concentration in all the ICU patients. In particular, plasma clusterin concentrations were significantly lower in patients with sepsis (n = 133, median 48.9 µg/mL, range 4.8–144.1 µg/mL) compared with patients without sepsis (n = 67, median 60.0 µg/mL, range 17.7–112.9 µg/mL; Figure 1B). These observations are consistent with findings from previous reported studies that decreased clusterin levels are found in patients with a systemic inflammatory response such as sepsis or septic shock [39,40].

### 3.2. Clusterin Is Not Associated with Age, Sex, Disease Etiology or Severity, Vasopressor Therapy, or Mechanical Ventilation

In our study cohort, typical sepsis-related infection sites were pneumonia, abdomen, and genitourinary tract, while non-sepsis-related causes of critical illness included cardiopulmonary disease, acute pancreatitis, and decompensated liver cirrhosis (Table 2). However, among critically ill patients, there was no consistent correlation between clusterin plasma concentrations and the various disease etiologies resulting in ICU admission (all *p* > 0.05; data not shown).

Although clusterin has associations to various systemic inflammatory, metabolic, and neurological disorders [28,29,30,31], clusterin plasma levels were not associated with established clinical disease severity scores, such as in critically ill patients with a high Acute Physiology And Chronic Health Evaluation-II (APACHE-II) score above 10 (*p* = 0.960) (Figure 1C). Additionally, vasopressor therapy did not significantly alter plasma clusterin concentrations in ICU patients (data not shown). Furthermore, there was also no significant difference in plasma clusterin levels between ventilated or non-ventilated critically ill patients (*p* = 0.602) (Figure 1D).

**Figure 2:**
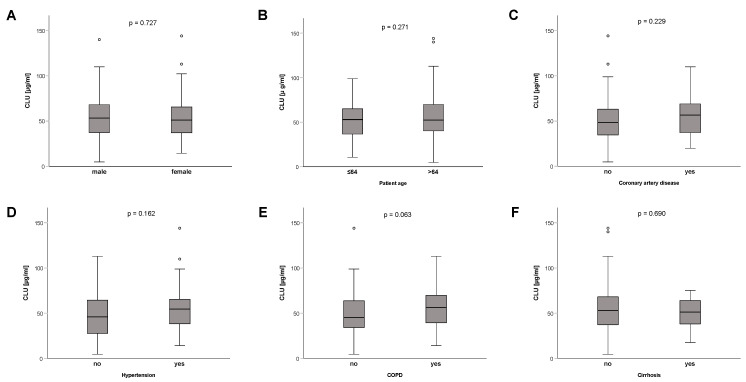
Clusterin levels are not influenced by sex, age, or comorbidities. Clusterin plasma concentrations were not influenced by patient sex (**A**), age (**B**), or pre-existing chronic diseases, such as coronary artery disease (**C**), hypertension (**D**), COPD (**E**), or liver cirrhosis (**F**). Circle depicts outlier.

Clusterin concentrations did not differ significantly between male and female patients (*p* = 0.727) (Figure 2A), patients aged up to 64 years or older (*p* = 0.271) (Figure 2B), or patients with or without certain comorbidities, such as: coronary artery disease (*p* = 0.229) (Figure 2C), hypertension (*p* = 0.162) (Figure 2D), chronic obstructive pulmonary disease (COPD) (*p* = 0.063) (Figure 2E), or liver cirrhosis (*p* = 0.690) (Figure 2F). Patients with active malignant diseases (n = 27) showed a tendency towards lower clusterin levels; however, the differences were not significant (*p* = 0.560); data not shown.

### 3.3. Association of Clusterin Plasma Concentrations at ICU Admission with Metabolic Alterations and Inflammatory Response

**Figure 3:**
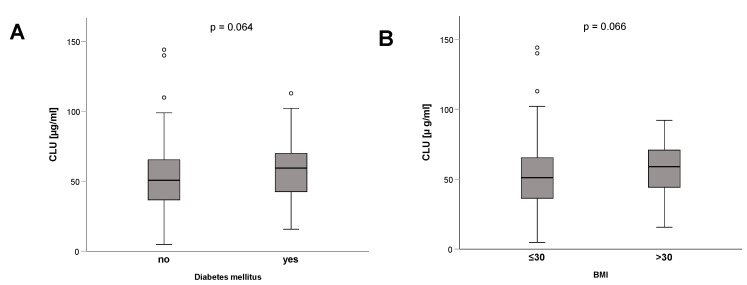
Impact of metabolic comorbidities on clusterin levels. Clusterin plasma concentrations did not differ between ICU patients with or without pre-existing type 2 diabetes (**A**) and obesity, as defined by a body mass index (BMI) above 30 kg/m^2^ (**B**). *p*-values (U-test) are given in the figure. Circle depicts outlier.

**Figure 4:**
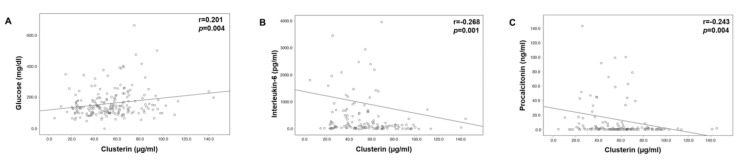
Clusterin levels correlate with glucose as well as inversely with inflammatory parameters. Correlation analyses revealed associations between plasma clusterin and plasma glucose (**A**), interleukin-6 (**B**), and procalcitonin (**C**), respectively. *p*-values (U-test) are given in the figure.

Clusterin has been linked to metabolic [27] and inflammatory diseases [10,12,13,14,15], as well as hepatic function [23]. Accordingly, we investigated the impact of metabolic alterations, including pre-existing obesity or diabetes, on clusterin concentrations in ICU patients. Interestingly, patients with pre-existing type 2 diabetes had numerically higher clusterin concentrations (median 59.5 µg/mL in diabetics vs. median 50.8 µg/mL in non-diabetics, *p* = 0.064; Figure 3A). This result is consistent with the positive association between clusterin and blood glucose (Table 3, Figure 4A). Patients with obesity defined by a body mass index above 30 kg/m² tended to have higher clusterin levels (median 58.9 µg/mL vs. median 51.1 µg/mL in non-obese patients, *p* = 0.066) (Figure 3B).

Regarding a possible association between clusterin and inflammation, we showed that clusterin was inversely associated with interleukin-6 (Figure 4B) and procalcitonin (Figure 4C). These findings are consistent with the lower clusterin concentrations in ICU patients with vs. without sepsis (Table 3). In these patients, receiver operating curve (ROC) analysis showed a lower diagnostic sensitivity and specificity of clusterin for the detection of sepsis compared with interleukin-6 and procalcitonin (data not shown). Interestingly, on the one hand, elevated Clusterin concentrations in the ICU cohort were associated with more prominent coagulability based on global coagulation tests, such as the prothrombin time–international normalized ratio (INR) and partial thromboplastin time (PTT); however, on the other hand, increasing clusterin levels correlated with lower fibrinogen levels and higher ATIII levels, which represent a hypocoagulative state (Table 3).

### 3.4. Clusterin Plasma Concentrations at ICU Admission Have No Association with Short-Term or Long-Term Mortality

**Figure 5:**
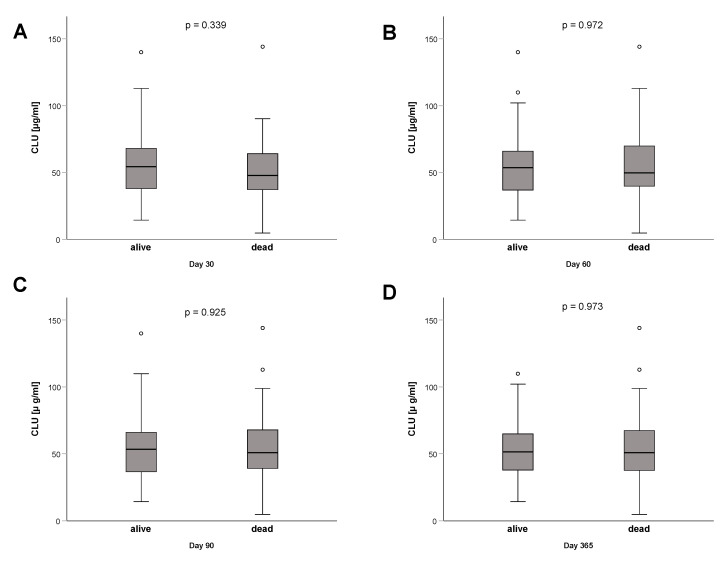
Clusterin is not a prognostic biomarker for mortality in critically ill patients. Clusterin plasma concentrations did not differ between survivors and non-survivors 30 days after ICU admission (**A**), 60 days after admission (**B**), 90 days after admission (**C**), or one year after admission (**D**), respectively. *p*-values (U-test) are given in the figure. ICU, intensive care unit. Circle depicts outlier.

To investigate a potential prognostic role of secreted clusterin in an ICU cohort, we compared baseline clusterin plasma concentrations of patients surviving and not surviving different time points. There was no significant difference in clusterin concentrations between survivors and non-survivors at 30 days after admission (*p* = 0.339) (Figure 5A), 60 days after admission (*p* = 0.972) (Figure 5B), 90 days after admission (*p* = 0.925) (Figure 5C), or one year after admission (*p* = 0.973) (Figure 5D), respectively. These findings suggest that clusterin cannot predict prognosis in critical illness.

## 4. Discussion

Multiple pathways are dysregulated in critical illness, as body homeostasis is challenged by catabolic stress responses and systemic inflammation upon life-threatening conditions. Based on the central role of clusterin as a regulator of humoral immunity and energy metabolism [6,7,8,9], we hypothesized that clusterin exposes a beneficial diagnostic performance in terms of disease severity, immunologic modulation, metabolic changes, and prediction of mortality in an ICU setting.

Clusterin is found in high- and low-density lipoproteins HDL and LDL. Clusterin in HDL is related to insulin sensitivity, whereas clusterin in LDL is closely correlated with insulin resistance [5]. Correspondingly, previous clinical data implicated that circulating clusterin (a) correlates closely with insulin resistance and (b) decreases in line with improving insulin sensitivity in type 2 diabetes [41]. Notably, evidence from experimental clusterin knockout animal models suggests that the liver is the predominant site of clusterin plasma concentration [42]. The liver is interconnected with several metabolic tissues such as adipose tissue, by this exertion of various cellular metabolic activities [42].

In this study, we demonstrated a trend to higher clusterin concentrations in diabetic ICU patients and a strong association between serum glucose and clusterin. Potentially, clusterin is released by the liver to improve insulin-dependent glucose uptake by muscles due to impaired metabolic function in critically ill patients [42]. Clusterin of hepatic origin is transported to skeletal muscle, where it binds to its cell surface receptor LRP2 (low-density lipoprotein receptor-related protein 2; megalin) to increase insulin action and to regulate glucose hemostasis, insulin signalling, and energy metabolism [43,44,45].

Although Hoofnagle and colleagues [5] found a negative relationship between the clusterin concentration in HDL and body mass index, we found a trend towards elevated clusterin levels in obese critically ill ICU patients, as well as a strong correlation between clusterin levels and triglycerides. As triglycerides are key components of the metabolic syndrome, elevated clusterin may indicate improved lipid transport and metabolism to preserve a metabolic homeostasis and cardioprotection [5]. Among obese patients, however, plasma levels of clusterin were found to be increased as well as directly related to obesity-related metabolic disease complications, among which were insulin resistance, dyslipidemia, and hepatic steatosis [24]. These results propose that peripheral-derived clusterin may play a novel role in the pathophysiology of critically ill patients, particularly in the context of coexisting metabolic impairment. In obesity characterized by a body mass index above 30 kg/m², clusterin concentrations are increased, and associated with insulin resistance and inflammatory markers [46].

Notably, Garcia-Obregon and colleagues found lowered clusterin concentrations in patients with sepsis [39]. Furthermore, DeCoux et al. reported that decreased clusterin is associated with mortality in critically ill septic patients [40]. In line with these previous findings, ICU patients with sepsis showed significantly lower clusterin levels than non-sepsis patients in our study. This finding corresponds with the inverse correlation of clusterin with proinflammatory markers such as interleukin-6, procalcitonin, or retinol binding protein-4. Moreover, for the first time, we were able to demonstrate that ICU patients in general (sepsis and non-sepsis) showed a significant increase of clusterin. Elevated clusterin and the inverse association with pro-inflammatory biomarkers may be the result of a clusterin-derived modulatory process to reduce hyperinflammation as a defensive mechanism to prevent cytokine storm and cell death [11,12,13,14,21].

## 5. Conclusions

In conclusion, our study, in a thoroughly documented cohort of ICU patients demonstrates elevated clusterin plasma concentrations as compared to healthy subjects. Moreover, we confirm decreased clusterin plasma concentrations in ICU patients with sepsis versus non-septic critically ill patients. Clusterin is not an indicator of disease severity, organ failure, or mortality; however, it is associated with metabolic and inflammatory alterations. We describe a challenging discordance between the key pathogenic function of clusterin in cellular stress and its weak capacity as a diagnostic clinical biomarker for predicting outcome in critical illness.

Therefore, prospective investigations will need to further decipher clusterin’s complex and distinct pathophysiological molecular functions in critically ill patients.

## Figures and Tables

**Table 1:** Basic parameters and characteristics of ICU patients.

Parameter	All Patients	Non-Sepsis	Sepsis	*p*-Value *
Number	200	67	133	
Sex (male/female)	122/78	44/23	78/55	0.360
Age median (range) [years]	64 (18–90)	62 (18–85)	65 (20–90)	0.612
APACHE-II score median (range)	18 (2–43)	14 (2–33)	19 (4–43)	0.002
SOFA score median (range)	9 (0–17)	8 (0–17)	9 (2–17)	0.055
SAPS-2 score median (range)	41 (0–73)	41 (13–72)	41.5 (0–73)	0.280
ICU days median (range)	7 (1–137)	6 (1–45)	9 (1–137)	0.013
Death during ICU n(%)	43 (21.5%)	9 (13.4%)	34 (25.6%)	0.067
Death during follow up (total) n(%)	78 (41.3%)	21 (33.3%)	57 (45.2%)	0.158
Mechanical ventilation n(%)	134 (67.7%)	46 (68.7%)	91 (44.8%)	0.915
Ventilation time median (range) [h]	116 (0–3628)	66 (0–3628)	123.5 (0–2966)	0.350
Vasopressor therapy n(%)	123 (61.5%)	33 (16.3%)	92 (45.3%)	0.011
Pre-existing diabetes n(%)	62 (31.6%)	22 (33.8%)	43 (32.1%)	0.061
BMI median (range) [m²/kg]	25.8 (15.3–86.5)	25.7 (15.9–40.5)	25.8 (15.3–86.5)	0.621
WBC median (range) [×10³/µL]	12.7 (0–208)	12.1 (1.8–29.6)	13.6 (0–208)	0.041
CRP median (range) [mg/dL]	96 (0–230)	17 (5–230)	154 (0–230)	0.001
Procalcitonin median (range) [µg/L]	0.6 (0–207.5)	0.2 (0.03–17.4)	1.8 (0–207.5)	0.001
Cystatin C median (range) [mg/dL]	1.6 (0–7.3)	1.17 (0.41–7.3)	2.06 (0–6.33)	0.001
GFR cystatin C median (range) [mL/min]	35 (0–379)	63 (5–379)	21.5 (0–218)	0.001
INR median (range)	1.16 (0–6.73)	1.16 (0.95–6.73)	1.16 (0–3.67)	0.975
PTT median (range) [s]	31 (0–150)	27.5 (20–150)	33 (0–150)	0.001
Clusterin day 1 median (range) [µg/mL]	52.9 (4.8–144.1)	60.0 (17.7–112.9)	48.9 (4.8–144.1)	0.026
ALT day 1 median (range) [U/L]	29.8 (0–6550)	36.5 (7–6550)	24.5 (0–5890)	0.034
AST day 1 median (range) [U/L]	41 (0–20,332)	49 (11–20,332)	39 (0–7832)	0.081
NT-pro-BNP day 1 median (range) [ng/L]	2353 (0–39,442)	476 (18–14,690)	3274 (0–39,442)	0.001

ALT, alanine aminotransferase; APACHE, Acute Physiology And Chronic Health Evaluation; AST, aspartate aminotransferase; BMI, body mass index; CRP, C-reactive protein; ICU, intensive care unit; INR, prothrombin time–international normalized ratio; NT-pro-BNP, N-terminal prohormone of brain natriuretic peptide; PTT, partial thromboplastin time; WBC, white blood cell. For quantitative variables, median and range (in parenthesis) are given. * *p*-values for the comparison of sepsis and non-sepsis patients are given (*U*-test for quantitative variables or chi-square test for qualitative parameters).

**Table 2:** Etiology of disease in the study population.

	Sepsis	Non-Sepsis
	n = 133	n = 67
**Etiology of sepsis critical illness** Site of infection n (%)		
pulmonary	68 (51.1%)	
abdominal	26 (19.1%)	
urogenital	8 (5.9%)	
other	31 (22.8%)	
**Etiology of non-sepsis critical illness** n (%)		
cardio-pulmonary disorder		28 (41.8%)
acute pancreatitis		9 (13.4%)
acute liver failure		3 (4.5%)
decompensated liver cirrhosis		8 (11.9%)
severe gastrointestinal hemorrhage		4 (6.0%)
non-sepsis other		15 (22.4%)

**Table 3:** Correlations of laboratory parameters with clusterin plasma concentrations (Spearman rank correlation test; only significant results are shown).

	ICU Patients	
Parameters	r	*p*
*Markers of inflammatory response*		
IL-6	−0.268	0.001
PCT	−0.243	0.004
*Markers of coagulation*		
Prothrombin time	0.183	0.011
INR	−0.171	0.017
PTT	−0.243	0.001
Fibrinogen	−0.241	0.007
AT III	0.242	0.007
*Markers of organ function*		
Bilirubin conjugated	−0.315	0.001
PCHE	0.19	0.011
Albumin	0.296	0.002
Protein	0.218	0.005
Lipase	−0.203	0.011
*Marker of diabetes*		
Glucose	0.201	0.004
*Markers of lipid metabolism*		
Triglycerides	0.237	0.003
Total cholesterol	0.288	<0.001
LDL cholesterol	0.384	0.002
HDL cholesterol	0.292	0.02
*Adipocytokines/metabolic markers*		
RBP4	0.336	0.007
Myostatin	0.261	0.037
Sclerostin	0.287	<0.001

AT III, antithrombin III; HDL cholesterol, high density lipoprotein cholesterol; IL-6 interleukin-6; INR, prothrombin time–international normalized ratio; LDL cholesterol, high density lipoprotein cholesterol; PCHE, pseudocholinesterase; PCT, procalcitonin; PTT, partial thromboplastin time; RBP4, retinol binding protein 4.

## Data Availability

The data presented in this study are available on request from the corresponding author.
